# Supplementary material for: EXSEQREG: Explaining sequence-based NLP tasks with regions with a case study using morphological features for named entity recognition
Source: PLoS One. 2020 Dec 30;15(12):e0244179. doi: 10.1371/journal.pone.0244179 (PMC7773252; doi:10.1371/journal.pone.0244179)
Supplement: Attachment — Submitted filename: Response to reviewers 20200916-1445.pdf [file pone.0244179.s002.pdf]

Response to Reviewers
EXSEQREG: Explaining sequence-based NLP tasks with regions: With a case study using morphological features for named entity recognition [manuscript id: PONE-D-19-26331]

Onur Güngör, Tunga Güngör, Suzan Uskudarli

Journal: *PLOS ONE*

Dear Editor,

Our last review found our manuscript technically acceptable, however requested a few editorial corrections. We have addressed all of these corrections. Furthermore, we have meticulously reviewed the entire manuscript to improve its grammar and narration. This review was performed by each other as well as an external person. This revision focuses solely on improving the clarity, accessibility and linguistic quality of our manuscript. The scope and technical content of this submission remains the same as the previous version.

We provide a point-to-point response to each reviewer.

Regards,
The authors

16/09/2020

Point-to-Point responses

The detailed point-to-point responses to the issues identified by the reviewers are provided below.

Reviewer #1:

Dear Reviewer,

We thank you for reviewing our submission and the valuable input and suggestions you provided to improve our manuscript. We have attempted to respond to all the issues and suggestions raised in your review.

In addition to addressing your suggestions, we made several editorial changes to improve the clarity and flow of the manuscript. The scope and technical content of our manuscript has not been altered. In other words, our editorial modifications have been performed only to improve the clarity, accessibility and linguistic quality of our work. The file labeled as 'Revised Manuscript with Track Changes' is annotated with special markers so that the additions are written in red underlined text while the deletions are written in blue text that is crossed out.

The following are point to point responses to your review:

Comment 1:

Same research was published -> Some research was published

in a given region, . -> in a given region.

impact of each feature to the current prediction -> impact of each feature on the current prediction

can be referred as an explanation -> can be referred to as an explanation

Only for the precision metric of ..., the model's performance is affected -> Only for the precision metric of ..., is the model's performance affected

a relatively less number of co-occurring morphological tags -> a relatively smaller number of co-occurring morphological tags

Response 1:

Thank you for your corrections. We have corrected all of the errors you have pointed out. Furthermore, we have performed a full review of the paper from an editorial perspective. We have addressed all the grammatical issues identified during this review. Also, we have modified the manuscript to improve the clarity and narrative of our presentation. We believe that these efforts have significantly improved the quality of our presentation.

Reviewer #3:

Dear Reviewer,

We thank you for reviewing our submission and the valuable input and suggestions you provided to improve our manuscript. In this revision we have made several editorial changes to improve the clarity and flow of the manuscript. The scope and technical content of our manuscript has not been altered. In other words, our editorial modifications have been performed only to improve the clarity, accessibility and linguistic quality of our work. The file labeled as 'Revised Manuscript with Track Changes' is annotated with special markers so that the additions are written in red underlined text while the deletions are written in blue text that is crossed out.

The following are point to point responses to your review:

Comment 1:

This version of the paper is much better than the previous one. I strongly support its publishing in the journal.

Response 1:

We are very pleased and encouraged by your comment. Thank you for your recommendation.
